# Atrial Fibrillation and Clinical Outcomes Following Mitral Transcatheter Edge-to-Edge Repair According to Mitral Regurgitation Etiology

**DOI:** 10.1016/j.jacasi.2026.02.028

**Published:** 2026-05-06

**Authors:** Tetsu Tanaka, Masaki Izumo, Shingo Kuwata, Taishi Okuno, Daisuke Miyahara, Yoshihiro J. Akashi, Masanori Yamamoto, Shunsuke Kubo, Mike Saji, Yuki Izumi, Yusuke Enta, Shinichi Shirai, Shingo Mizuno, Yusuke Watanabe, Makoto Amaki, Kazuhisa Kodama, Junichi Yamaguchi, Toru Naganuma, Hiroki Bota, Yohei Ohno, Masahiko Asami, Daisuke Hachinohe, Masahiro Yamawaki, Hiroshi Ueno, Kazuki Mizutani, Toshiaki Otsuka, Kentaro Hayashida, OCEAN-Mitral Investigators

**Affiliations:** a Department of Cardiology, St Marianna University School of Medicine, Kawasaki, Japan; b Department of Cardiology, Toyohashi Heart Center, Toyohashi, Japan; c Department of Cardiology, Gifu Heart Center, Gifu, Japan; d Department of Cardiology, Nagoya Heart Center, Nagoya, Japan; e Department of Cardiology, Kurashiki Central Hospital, Kurashiki, Japan; f Department of Cardiology, Sakakibara Heart Institute, Tokyo, Japan; g Division of Cardiovascular Medicine, Department of Internal Medicine, Toho University Faculty of Medicine, Tokyo, Japan; h Department of Cardiology, Sendai Kosei Hospital, Sendai, Japan; i Division of Cardiology, Kokura Memorial Hospital, Kitakyushu, Japan; j Department of Cardiology, Shonan Kamakura General Hospital, Kanagawa, Japan; k Department of Cardiology, Teikyo University School of Medicine, Tokyo, Japan; l Department of Cardiology, National Cerebral and Cardiovascular Center, Suita, Japan; m Division of Cardiology, Saiseikai Kumamoto Hospital Cardiovascular Center, Kumamoto, Japan; n Department of Cardiology Tokyo Woman’s Medical University, Tokyo, Japan; o Department of Cardiology, New Tokyo Hospital, Chiba, Japan; p Department of Cardiology, Sapporo Higashi Tokushukai Hospital, Sapporo, Japan; q Department of Cardiology, Tokai University School of Medicine, Isehara, Japan; r Division of Cardiology, Mitsui Memorial Hospital, Tokyo, Japan; s Department of Cardiology, Sapporo Heart Center, Sapporo Cardiovascular Clinic, Sapporo, Japan; t Department of Cardiology, Saiseikai Yokohama City Eastern Hospital, Kanagawa, Japan; u Second Department of Internal Medicine, Toyama University Hospital, Toyama, Japan; v Division of Cardiology, Department of Medicine, Kindai University Faculty of Medicine, Osaka, Japan; w Department of Hygiene and Public Health, Nippon Medical School, Tokyo, Japan; x Department of Cardiology, Keio University School of Medicine, Tokyo, Japan

**Keywords:** atrial fibrillation, etiology of mitral regurgitation, mitral regurgitation, transcatheter edge-to-edge repair

## Abstract

**Background:**

The association between atrial fibrillation (AF) and prognosis after mitral transcatheter edge-to-edge repair (M-TEER) remains unclear.

**Objectives:**

The authors examined the association between AF and clinical outcomes after M-TEER according to the etiology of mitral regurgitation (MR).

**Methods:**

A total of 3,764 patients were classified into degenerative mitral regurgitation (DMR), ventricular functional mitral regurgitation (VFMR), and atrial functional mitral regurgitation (AFMR). We further stratified the patients by the presence of AF. The primary outcome was all-cause mortality within 2 years.

**Results:**

The prevalence of AF was 57.4% (n = 646 of 1,126) in DMR, 59.7% (n = 1,319 of 2,211) in VFMR, and 83.8% (n = 358 of 427) in AFMR. During 2-year follow-up (the median follow-up: 427 [IQR: 301-821] days), 660 of 3,764 patients (17.5%) died. The association between AF and all-cause mortality had a significant interaction with MR etiology (*P* for interaction <0.001). AF was associated with a higher risk of all-cause mortality in DMR (adjusted HR: 1.88; 95% CI: 1.17-3.02; *P* = 0.009), whereas this association was not significant in VFMR and AFMR. Among patients with VFMR, left atrial (LA) volume index modified the association between AF and all-cause mortality. AF was related to a higher risk of all-cause mortality in VFMR patients with lower LA volume index, whereas this association was attenuated in those with higher LA volume index.

**Conclusions:**

AF was associated with all-cause mortality after M-TEER in patients with DMR, but not in those with VFMR or AFMR. The prognostic effect of AF in patients undergoing M-TEER may be modulated by MR etiology and underlying LA remodeling.

Mitral regurgitation (MR) is a prevalent valvular heart disease that affects the prognosis of patients.^1^ MR is classified into 3 distinct etiologies based on the underlying pathophysiological mechanism: degenerative MR (DMR), ventricular functional MR (VFMR), and atrial functional MR (AFMR).^2^ These 3 etiologies not only have different cardiac function and geometry but also present distinct clinical outcomes.^3^

Atrial fibrillation (AF) is one of the most common arrhythmias in patients with MR and is closely related to the development and progression of MR.^4^^,^^5^ In patients with DMR, progressive MR leads to left atrial (LA) remodeling and contributes to the development of AF. The presence of AF can worsen hemodynamics of MR and further accelerate the LA remodeling due to DMR. On the other hand, VFMR and AFMR both result from left ventricular (LV) or LA dilation, and AF can contribute to the development of functional MR. These functional subtypes of MR often present advanced stages of LA or LV remodeling. Mitral transcatheter edge-to-edge repair (M-TEER) is an emerging treatment option for symptomatic MR. M-TEER is safe and effective in reducing MR, irrespectively of MR etiologies.^6^, ^7^, ^8^, ^9^ Several studies have investigated the clinical implication of AF in patients undergoing M-TEER^10^, ^11^, ^12^, ^13^; however, given the differences in underlying pathophysiology and cardiac function, the clinical implication of AF after M-TEER might differ between the MR etiologies. In this study, we therefore investigated the association between AF and clinical outcomes after M-TEER in each etiology of MR.

## Methods

### Study population

The OCEAN (Optimized CathEter vAlvular iNtervention)-Mitral registry is an ongoing, prospective, investigator-initiated, multicenter observational registry in patients with MR undergoing M-TEER.^14^^,^^15^ A total of 21 Japanese centers participated in this registry. OCEAN-Mitral operates independently of industrial influence. This registry included 3,764 patients who underwent M-TEER with the MitraClip G2 or G4 system (Abbott Vascular Inc) from April 2018 to June 2023. Patient characteristics, procedural details, and clinical outcomes are systematically recorded. Each case was reviewed before M-TEER by a multidisciplinary heart valve team consisting of interventional cardiologists, cardiac surgeons, echocardiologists, and heart failure specialists. The indication for M-TEER in DMR was a high surgical risk, whereas for AFMR or VFMR, it was symptomatic status despite optimal medical therapy and, if applicable, cardiac resynchronization therapy. Treatment indications followed Japanese guidelines.^16^

Informed consent was obtained from all patients, and the study was approved by the relevant institutional review board and conducted in accordance with the Declaration of Helsinki principles. The study is registered with the University Hospital Medical Information Network (UMIN000023653).

### Data collection

Clinical, echocardiographic, and laboratory data were prospectively collected at prespecified timing (before and after the procedure, at 1 month and 1 year after the procedure, and annually thereafter). All information was prospectively collected and reported by the investigators. Follow-up assessments were performed via clinical visits and/or telephone contacts. When necessary, the referring cardiologists, general practitioners, and patients were contacted to obtain further information.

### Echocardiographic assessments

All the echocardiographic assessments were performed by experienced echocardiographers according to current guidelines.^17^^,^^18^ The severity of MR was evaluated based on qualitative and quantitative criteria and was classified as 0 (none/trivial), 1+ (mild), 2+ (moderate), 3+ (moderate to severe), or 4+ (severe). The etiology of MR was classified into degenerative or functional MR. LA volume index was calculated as the maximum LA volume indexed to body surface area, with LA volume assessed using the Simpson’s biplane method.^17^ According to the current guidelines,^17^ each echocardiographic parameter was reported as the average of 3 consecutive beats in patients without AF and 5 beats in those with AF, or as a measurement using a representative single beat when averaging was not feasible. Functional MR was further classified into AFMR and VFMR. AFMR was defined as cases that met all of the following criteria: 1) LV ejection fraction >50%; 2) no or mild LV enlargement (LV end-diastolic volume index ≤89 mL/m^2^ for men and ≤70 mL/m^2^ for women); and 3) moderate or severe LA enlargement (LA volume index ≥42 mL/m^2^).^19^^,^^20^ Patients lacking any of these criteria were considered to have ventricular functional MR.

### Outcome measures

The primary outcome was all-cause mortality within 2 years after M-TEER. We examined each of cardiovascular mortality, heart failure hospitalization, and ischemic stroke or transient ischemic attack within 2 years as secondary outcomes. Clinical events, including mortality and heart failure hospitalization, were routinely collected and independently adjudicated by local investigators based on the criteria of the Mitral Valve Academic Research Consortium.^21^

### Statistics

Continuous variables are presented as the mean ± SD or the median with an IQR and were compared with the Student's *t*-test or the Mann-Whitney *U* test, and categorical data are presented as numbers with percentages. Categorical data are presented as numbers with percentages and were compared by chi-square or Fisher’s exact test. Time-to-event curves are depicted using the Kaplan-Meier method and compared between groups using the log-rank test. Univariate and multivariable Cox proportional hazard models were used to calculate HRs and corresponding 95% CIs. HRs were adjusted for prespecified variables: age, male sex, body mass index, diabetes mellitus, hypertension, clinical frailty scale ≥IV, a prior history of stroke, chronic obstructive pulmonary disease, cardiac implantable electronic device, heart failure hospitalization within 1 year, use of anticoagulants, use of loop diuretic, NYHA functional class III or IV, estimated glomerular filtration rate ≤45 mL/m^2^/min, LV ejection fraction ≤40%, LV end-diastolic volume index ≤70 mL/m^2^, tricuspid annular plane systolic excursion <17 mm, LA volume index, tricuspid regurgitation ≥ moderate, systolic pulmonary artery pressure, mean transmitral valve gradient ≥5 mm Hg at discharge, and residual MR ≥ moderate at discharge. Multiple imputation was applied to values included in this multivariable model that had missing values. Detailed imputation procedures are provided in the Supplemental Methods and Supplemental Tables 1 to 3. We also conducted a complete-case analysis as a sensitivity analysis. The proportional hazards assumption was examined using Schoenfeld residuals; global tests showed no evidence of violations. Multiplicative interaction analyses were performed to examine potential effect modification of the association between AF and the primary outcome by MR etiology and LA volume index, given their established pathophysiological relevance to LA remodeling and prognosis. Interaction terms were constructed by including cross-product terms between AF and the aforementioned variables in Cox proportional hazards models, and their statistical significance was assessed using the Wald test. We depicted restricted cubic spline curves to model the correlation between AF and the primary outcome across LA volume index. The number of knots was determined according to Harrell’s recommended approach, and the knots were placed at the 10th, 50th, and 90th percentiles of LA volume index. In addition, we conducted sensitivity analyses wherein patients were categorized according to subtypes of AF (paroxysmal or persistent/permanent AF) and atrial rhythm on the electrocardiogram before the M-TEER procedures (sinus rhythm, AF, or pacing rhythm). Statistical significance was set as a 2-sided *P* < 0.05. All analyses were conducted using Stata/SE 15.1 (StataCorp).

## Results

### Study population

All 3,764 patients of the OCEAN-Mitral registry were included in the present analysis and were stratified by MR etiology: 1,126 with DMR (29.9%), 2,211 with VFMR (58.7%), and 427 with AFMR (11.3%) (Supplemental Figure 1). Overall, 2,323 of the 3,764 patients (61.7%) had a history of AF. The prevalence of AF was 57.4% in DMR (n = 646 of 1,126), 59.7% in VFMR (n = 1,319 of 2,211), and 83.8% in AFMR (n = 358 of 427).

Baseline characteristics are shown in Table 1. Across the MR etiologies, patients with AF were older and were more likely to use anticoagulants than those without AF. Among patients with DMR, those with AF were more likely to have severe heart failure symptoms (ie, NYHA functional class III or IV) and multiple comorbidities compared with those without AF, resulting in a higher risk for mitral valve surgery. In patients with VFMR, those with AF were more likely to have frailty. Coronary artery disease was less common in patients with AF among VFMR and AFMR.

**Table 1:** Baseline Characteristics of the Patients

	DMR			VFMR			AFMR		
	AF (n = 646)	No AF (n = 480)	*P* Value	AF (n = 1,319)	No AF (n = 892)	*P* Value	AF (n = 358)	No AF (n = 69)	*P* Value
Age, y	83.6 ± 6.7	81.9 ± 9.5	<0.001	77.2 ± 8.9	74.2 ± 10.9	<0.001	82.3 ± 6.6	83.1 ± 6.1	0.36
Male	316 (48.9)	190 (39.6)	0.002	834 (63.2)	539 (60.4)	0.18	161 (45.0)	26 (37.7)	0.26
Body mass index, kg/m^2^	20.8 ± 3.3	21.2 ± 3.7	0.11	21.5 ± 3.5	21.1 ± 3.4	0.010	21.4 ± 3.5	21.4 ± 4.0	0.96
NYHA functional class III or IV	406 (62.8)	261 (54.4)	0.004	886 (67.2)	571 (64.0)	0.12	204 (57.0)	44 (63.8)	0.30
Prior HF hospitalization	515 (79.8)	307 (64.0)	<0.001	1169 (88.9)	780 (87.4)	0.30	291 (81.5)	53 (76.8)	0.36
Clinical frailty scale ≥4	399 (64.0)	244 (52.0)	<0.001	699 (55.3)	408 (47.3)	<0.001	204 (58.8)	39 (59.1)	0.96
STS score for mitral valve replacement, %	9.4 (6.3-14.2)	7.4 (4.9-10.9)	<0.001	9.4 (6.0-14.5)	8.8 (5.0-14.5)	0.006	9.0 (6.0-13.6)	10.5 (7.3-13.4)	0.42
Systolic blood pressure, mm Hg	111.6 ± 18.4	119.8 ± 18.7	<0.001	106.4 ± 17.6	106.9 ± 18.3	0.51	114.4 ± 19.0	121.1 ± 21.4	0.009
Diastolic blood pressure, mm Hg	64.9 ± 12.8	67.0 ± 12.2	0.004	63.6 ± 12.5	62.2 ± 12.2	0.006	65.4 ± 13.6	63.5 ± 13.7	0.28
Heart rate, beats/min	76.7 ± 17.2	71.6 ± 13.4	<0.001	75.7 ± 16.4	73.8 ± 14.1	0.005	73.2 ± 14.9	68.5 ± 12.4	0.016
CIED	44 (6.8)	26 (5.4)	0.34	401 (30.4)	252 (28.3)	0.28	60 (16.8)	9 (13.0)	0.44
Medical history									
Diabetes mellitus	85 (13.2)	71 (14.8)	0.43	434 (32.9)	308 (34.5)	0.43	77 (21.5)	13 (18.8)	0.62
Hypertension	435 (67.3)	346 (72.1)	0.088	819 (62.1)	545 (61.1)	0.64	249 (69.6)	58 (84.1)	0.014
Coronary artery disease	123 (19.0)	95 (19.8)	0.75	525 (39.8)	477 (53.5)	<0.001	64 (17.9)	27 (39.1)	<0.001
Cardiac surgery	65 (10.1)	30 (6.2)	0.023	151 (11.4)	87 (9.8)	0.21	35 (9.8)	10 (14.5)	0.24
Stroke	82 (12.7)	47 (9.8)	0.13	161 (12.2)	89 (10.0)	0.10	46 (12.8)	6 (8.7)	0.33
COPD	57 (8.8)	45 (9.4)	0.75	122 (9.2)	67 (7.5)	0.15	27 (7.5)	6 (8.7)	0.74
Dialysis	19 (2.9)	16 (3.3)	0.71	84 (6.4)	98 (11.0)	<0.001	10 (2.8)	2 (2.9)	0.96
Liver cirrhosis	10 (1.5)	6 (1.2)	0.68	19 (1.4)	19 (2.1)	0.22	7 (2.0)	1 (1.4)	0.78
Hemoglobin, g/dL	11.6 ± 1.7	11.6 ± 1.7	0.72	11.8 ± 1.9	11.7 ± 1.9	0.24	11.3 ± 1.7	11.0 ± 1.8	0.18
eGFR, mL/min/1.73 m^2^	39.3 (28.2-51.0)	46.8 (31.3-59.3)	<0.001	35.2 (24.5-47.6)	35.6 (21.3-49.8)	0.69	37.5 (28.5-48.4)	36.7 (21.2-44.0)	0.047
eGFR <60 mL/min/1.73 m^2^	575 (89.3)	368 (76.7)	<0.001	1172 (89.5)	772 (87.5)	0.15	309 (86.3)	64 (92.8)	0.17
eGFR <45 mL/min/1.73 m^2^	410 (63.7)	222 (46.3)	<0.001	923 (70.5)	591 (67.0)	0.082	242 (67.6)	52 (75.4)	0.20
NT-proBNP, pg/mL	2,383 (1,178-4,817)	743 (333- 1,818)	<0.001	3,310 (1,786-6,829)	3,610 (1,687-7,367)	0.61	1,980 (822-3,867)	1,970 (1,000-3,605)	0.75
Type of AF/AFL									
Paroxysmal	256 (30.6)			489 (32.1)			87 (24.3)		
Persistent or permanent	390 (60.4)			830 (62.9)			271 (75.7)		
Medical treatment									
ACEI/ARB/ARNI	377 (58.4)	305 (63.5)	0.078	843 (63.9)	614 (68.8)	0.017	213 (59.5)	41 (59.4)	0.99
Beta-blockers	417 (66.4)	211 (44.8)	<0.001	1,082 (83.1)	735 (83.1)	0.98	237 (67.3)	55 (79.7)	0.041
MRA	338 (52.3)	183 (38.1)	<0.001	827 (62.8)	508 (57.1)	0.007	188 (53.0)	24 (35.3)	0.008
SGLT2i	71 (11.0)	42 (8.8)	0.22	367 (27.8)	243 (27.2)	0.76	57 (15.9)	5 (7.2)	0.061
Loop diuretics	530 (82.0)	322 (67.1)	<0.001	1,105 (83.8)	687 (77.0)	<0.001	306 (85.5)	53 (76.8)	0.072
Antiplatelets	120 (18.6)	250 (52.1)	<0.001	391 (29.6)	575 (64.5)	<0.001	64 (17.9)	39 (56.5)	<0.001
Anticoagulants	583 (90.2)	213 (44.4)	<0.001	1,172 (88.9)	476 (53.4)	<0.001	323 (90.2)	39 (56.5)	<0.001

Values are n (%), mean ± SD, or median (IQR).

ACEI = angiotensin-converting enzyme inhibitor; AF = atrial fibrillation; AFL = atrial flutter; AFMR = atrial functional mitral regurgitation; ARB = angiotensin receptor blocker; ARNI = angiotensin receptor/neprilysin inhibitor; CIED = cardiac implantable electronic device; COPD = chronic obstructive pulmonary disease; DMR = degenerative mitral regurgitation; eGFR = estimated glomerular filtration rate; HF = heart failure; MRA = mineralocorticoid receptor antagonist; NT-proBNP = N-terminal pro–B-type natriuretic peptide; SGLT2i = sodium-glucose cotransporter 2 inhibitor; STS = Society of Thoracic Surgeons; VFMR = ventricular functional mitral regurgitation.

Echocardiographic findings are shown in Table 2. MR severity was comparable between patients with and without AF in each MR etiology. Across all MR etiology, patients with AF had larger LA volume index, lower right ventricular systolic function, and higher prevalent of moderate or greater tricuspid regurgitation than those without AF. Patients with AF had lower LV ejection fraction in the DMR group but higher LV ejection fraction in the VFMR group, compared with those without AF. LV volumes were smaller in patients with AF compared with those without AF in both DMR and VFMR. Mitral valve area was larger in patients with AF in both VFMR and AFMR.

**Table 2:** Preprocedural, Procedural, and Postprocedural Findings

	DMR			VFMR			AFMR		
	AF (n = 646)	No AF (n = 480)	*P* Value	AF (n = 1,319)	No AF (n = 892)	*P* Value	AF (n = 358)	No AF (n = 69)	*P* Value
Preprocedural echocardiographic findings									
LVEF, %	58.7 ± 11.0	62.0 ± 11.4	<0.001	36.3 ± 11.8	32.0 ± 9.7	<0.001	60.3 ± 6.2	60.5 ± 7.5	0.97
LVEF category			0.19			<0.001			–
LVEF <40%	54 (8.4)	36 (7.5)		861 (65.4)	732 (82.1)		0 (0)	0 (0)	
LVEF 40% to <50%	55 (8.5)	28 (5.8)		310 (23.5)	120 (13.5)		0 (0)	0 (0)	
LVEF ≥50%	536 (83.1)	416 (86.7)		146 (11.1)	40 (4.5)		358 (100.0)	69 (100.0)	
LVEDVi, mL/m^2^	74.7 ± 25.9	81.8 ± 28.4	<0.001	107.5 ± 40.3	122.9 ± 44.7	<0.001	57.4 ± 14.1	58.8 ± 13.3	0.44
LVESVi, mL/m^2^	32.6 ± 17.7	33.9 ± 21.6	0.31	71.3 ± 34.9	87.2 ± 40.0	<0.001	23.3 ± 7.1	24.5 ± 7.1	0.25
LA volume index, mL/m^2^	98.7 ± 59.7	71.7 ± 28.4	<0.001	97.0 ± 58.7	66.7 ± 27.2	<0.001	111.1 ± 68.4	73.4 ± 41.5	<0.001
E/e’	17.2 ± 9.5	17.8 ± 6.5	0.24	17.1 ± 7.4	19.1 ± 7.5	<0.001	14.4 ± 5.7	15.9 ± 6.7	0.12
MR severity at rest			0.38			0.085			0.32
Moderate or less	35 (5.4)	25 (5.2)		196 (14.9)	165 (18.5)		60 (16.8)	17 (24.6)	
Moderate to severe	125 (19.3)	74 (15.4)		397 (30.1)	271 (30.4)		136 (38.0)	21 (30.4)	
Severe	486 (75.2)	381 (79.4)		726 (55.0)	456 (51.1)		162 (45.3)	31 (44.9)	
ERO, cm^2^	0.4 (0.3-0.6)	0.4 (0.3-0.6)	0.88	0.3 (0.2-0.4)	0.3 (0.2-0.4)	0.010	0.3 (0.2-0.4)	0.3 (0.2-0.4)	0.89
MVA, cm^2^	5.2 ± 1.5	5.2 ± 1.5	0.51	5.4 ± 1.7	5.0 ± 1.4	<0.001	5.1 ± 1.7	4.2 ± 1.4	<0.001
TMPG, mm Hg	2.0 (1.1-3.0)	2.0 (1.2-3.0)	0.60	1.5 (1.0-2.1)	1.5 (1.0-2.0)	0.61	1.6 (1.0-2.2)	1.6 (1.0-2.0)	0.78
TR ≥ moderate	326 (50.5)	128 (26.7)	<0.001	527 (40.0)	199 (22.3)	<0.001	195 (54.5)	18 (26.1)	<0.001
RVDd-base, mm	39.9 ± 8.3	37.3 ± 7.9	<0.001	41.0 ± 8.4	39.8 ± 8.9	0.021	40.1 ± 7.7	38.2 ± 8.1	0.19
RVDd-mid, mm	30.2 ± 6.7	29.1 ± 6.2	0.058	30.3 ± 6.9	30.3 ± 7.0	0.91	30.0 ± 7.0	30.7 ± 8.8	0.61
TAPSE, mm	17.2 ± 5.4	19.7 ± 4.4	<0.001	15.0 ± 4.6	16.6 ± 4.6	<0.001	16.1 ± 4.4	18.7 ± 4.6	<0.001
RVFAC, %	37.0 ± 9.4	40.2 ± 8.9	<0.001	34.0 ± 11.1	34.8 ± 11.6	0.25	39.2 ± 9.3	43.9 ± 8.8	0.006
SPAP, mm Hg	41.6 ± 14.3	39.2 ± 15.2	0.011	40.4 ± 14.7	40.6 ± 15.8	0.77	40.2 ± 13.6	34.6 ± 12.0	0.004
Procedural findings									
MitraClip G4 system	367 (56.8)	307 (64.0)	0.016	770 (58.4)	550 (61.7)	0.12	235 (65.6)	46 (66.7)	0.87
Number of implanted clips	1 (1-2)	1 (1-2)	0.031	1 (1-2)	1 (1-1)	0.001	1 (1-1)	1 (1-1)	0.42
Technical success	639 (98.9)	476 (99.2)	0.77	1,311 (99.4)	890 (99.8)	0.33	353 (98.6)	69 (100.0)	>0.99
Postprocedural findings									
MR severity at discharge			0.001			0.006			0.022
None/trivial	98 (15.3)	118 (24.6)		216 (16.6)	193 (21.8)		81 (22.9)	29 (42.0)	
Mild	341 (53.4)	245 (51.1)		790 (60.6)	530 (59.9)		197 (55.6)	29 (42.0)	
Moderate	158 (24.7)	88 (18.4)		253 (19.4)	145 (16.4)		70 (19.8)	10 (14.5)	
Moderate to severe	22 (3.4)	16 (3.3)		26 (2.0)	10 (1.1)		4 (1.1)	1 (1.4)	
Severe	20 (3.1)	12 (2.5)		18 (1.4)	7 (0.8)		2 (0.6)	0 (0.0)	
MR ≥ moderate at discharge	200 (31.0)	116 (24.2)	0.012	297 (22.5)	162 (18.2)	0.013	76 (21.2)	11 (15.9)	0.32
TMPG at discharge, mmHg	2.8 (2.0-3.8)	3.0 (2.0-4.0)	0.010	2.5 (1.9-3.5)	2.6 (2.0-3.9)	0.010	3.0 (2.0-3.9)	3.2 (2.0-4.7)	0.061
TMPG ≥5 mm Hg at discharge	56 (9.7)	64 (14.4)	0.021	109 (9.1)	91 (11.5)	0.092	35 (11.0)	14 (21.9)	0.017
In-hospital mortality	16 (2.5)	5 (1.0)	0.078	45 (3.4)	31 (3.5)	0.94	8 (2.2)	1 (1.4)	0.68

Values are n (%), mean ± SD, or median (IQR).

ERO = effective regurgitant orifice; LA = left atrium; LVEDVi = left ventricular end-diastolic volume index; LVEF = left ventricular ejection fraction; LVESVi = left ventricular end-systolic volume index; MR = mitral regurgitation; MVA = mitral valve area; RVDd = right ventricular diastolic diameter; RVFAC = right ventricular fractional area change; SPAP = systolic pulmonary artery pressure; TAPSE = tricuspid annular plane systolic excursion; TMPG = transmitral mean pressure gradient; TR = tricuspid regurgitation.

### Procedural outcomes

In 2,275 of 3,764 patients (60.4%), the M-TEER procedures were performed using MitraClip G4 systems. Overall, technical success rate was 99.3% (n = 3,738 of 3,764), and MR reduction to mild or less at discharge was achieved in 77.1% (n = 2,902 of 3,764). Patients with AF had a higher rate of residual MR ≥ moderate at discharge than those without AF, across all MR etiologies. In contrast, transmitral pressure gradient at discharge was likely to be lower in patients with AF. Among patients with DMR, those with AF were likely to have a higher rate of in-hospital mortality than those without AF, whereas the difference was not statistically significant.

### Clinical outcomes

**Figure 1:**
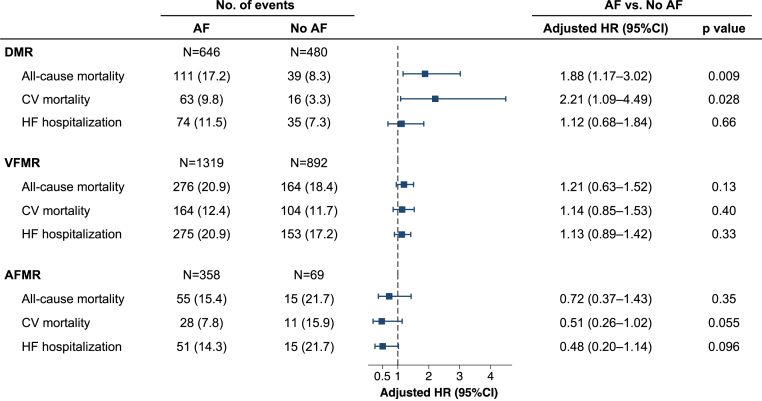
Association of AF With 2-Year Clinical Outcomes by Mitral Reguritation Etiology Association between atrial fibrillation (AF) and clinical outcomes, including all-cause mortality, cardiovascular (CV) mortality, and heart failure (HF) hospitalization, according to etiology of mitral regurgitation. Adjusted HRs and 95% CIs were calculated in multivariable models. AFMR = atrial functional mitral regurgitation; DMR = degenerative mitral regurgitation; VFM = ventricular functional mitral regurgitation.

The median follow-up length was 427 (IQR: 301-821) days. During the 2-year follow-up, 660 of the 3,764 patients (17.5%) died. The association between AF and 2-year all-cause mortality had a significant interaction with MR etiology (*P* for interaction <0.001). In the DMR group, patients with AF had a higher incidence of all-cause mortality within 2 years after M-TEER (Central Illustration). This association was consistent after adjusting for patient characteristics and procedural outcomes (adjusted HR: 1.80; 95% CI: 1.12-2.88; *P* = 0.015) (Figure 1). In contrast, AF was not associated with the risk of all-cause mortality in both VFMR and AFMR (adjusted HR: 1.21; 95% CI: 0.63-1.52; *P* = 0.12, and adjusted HR: 0.72; 95% CI: 0.37-1.43; *P* = 0.35, respectively).

**Central Illustration:**
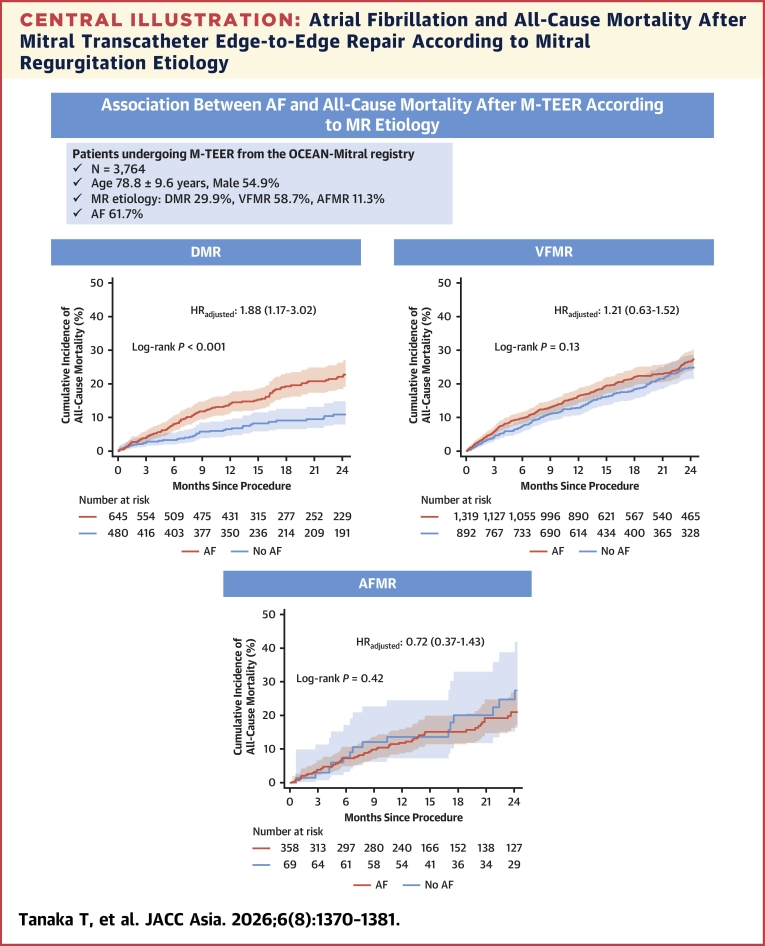
Atrial Fibrillation and All-Cause Mortality After Mitral Transcatheter Edge-to-Edge Repair According to Mitral Regurgitation Etiology Incidence of all-cause mortality after mitral transcatheter edge-to-edge repair (M-TEER) according to atrial fibrillation (AF) in patients with degenerative mitral regurgitation (DMR), ventricular functional mitral regurgitation (VFMR), and atrial functional mitral regurgitation (AFMR). Shading identifies 95% CI. OCEAN-Mitral = Optimized CathEter vAlvular iNtervention–Mitral Registry.

**Figure 2:**
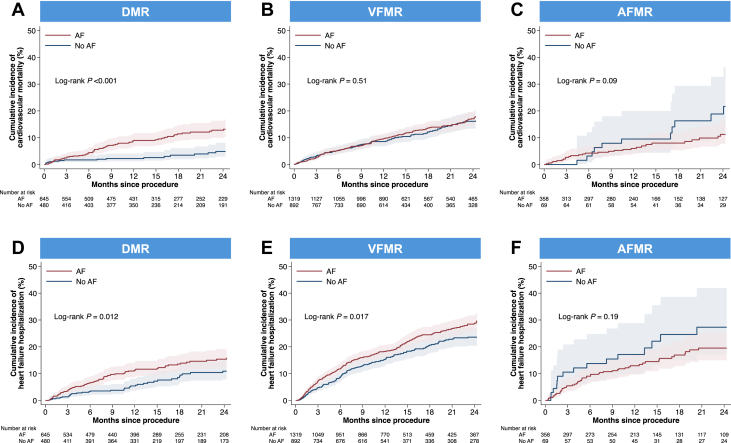
Cardiovascular Mortality and Heart Failure Hospitalization After M-TEER by Mitral Regurgitation Etiology Incidence of cardiovascular mortality (A to C) and heart failure hospitalization (D to F) after mitral transcatheter edge-to-edge repair (M-TEER) according to AF in patients with DMR (A and D), VFMR (B and E), and AFMR (C and F). Shading identifies 95% CI. Abbreviations as in Figure 1.

Also, AF was associated with a higher risk of cardiovascular mortality within 2 years after M-TEER in patients with DMR (adjusted HR: 2.07; 95% CI: 1.03-4.16; *P* = 0.041) (Figures 1 and 2). The risk of heart failure hospitalization was higher in patients with AF than those without AF among both DMR and VFMR, whereas this association was not significant after adjusting for patient characteristics and procedural results. In contrast, AF was associated with a lower risk of heart failure hospitalization in patients with AFMR (adjusted HR: 0.47; 95% CI: 0.24-0.92; *P* = 0.027). The rate of ischemic stroke or transient ischemic attack was comparable between patients with and without AF across the MR etiologies (Supplemental Figure 2). In a sensitivity analysis, the direction and magnitude of the effect estimates were consistent between the multiple imputation and complete-case analyses (Supplemental Table 4), supporting the robustness of the main findings.

We evaluated the severity of MR after the M-TEER procedures (Supplemental Figure 3). The rate of residual MR ≤2+ at 1- and 12-month follow-ups was comparable between patients with and without AF across all etiologies of MR.

### Interaction between AF and LA volume index

**Figure 3:**
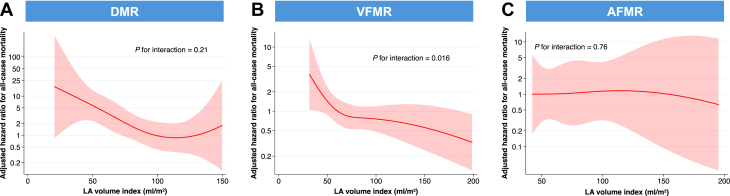
Association Between AF and All-Cause Mortality Modified by LA Volume Index Spline curves with 3 knots show the association between atrial fibrillation (AF) and 2-year all-cause mortality according to left atrial (LA) volume index in patients with DMR (A), VFMR (B), and AFMR (C). HRs were adjusted for the covariates included in the multivariable models. Abbreviations as in Figure 1.

Our spline curves show the trend of the association between AF and the primary outcome according to LA volume index (Figure 3). In patients with DMR, AF was consistently associated with a higher risk of 2-year mortality across LA volume index (*P* for interaction = 0.20). In contrast, the association between AF and the primary outcome was significantly modified by LA volume index in patients with VFMR (*P* for interaction = 0.016) and was more pronounced in those with a lower LA volume index.

### Sensitivity analysis

In sensitivity analyses, DMR patients with AF had a higher incidence of the primary outcome than those without AF across the subtypes of AF, paroxysmal or persistent/permanent AF (Supplemental Figure 4). On the other hand, any subtypes of AF were not associated with 2-year all-cause mortality in VFMR and AFMR.

Furthermore, we divided patients with AF into 3 groups based on atrial rhythm on electrocardiogram before the M-TEER procedure: sinus rhythm, AF rhythm, and pacing rhythm. DMR patients with AF had a higher incidence of the primary outcome across the types of atrial rhythm before the procedure (Supplemental Figure 5), whereas neither atrial rhythm was associated with the primary outcome in VFMR and AFMR patients.

## Discussion

In this large multicenter registry of patients undergoing M-TEER, we investigated the association between AF and clinical outcomes after M-TEER according to the etiology of MR. The main findings of the present study are as follows:

1. Approximately 60% of patients undergoing M-TEER had AF.
2. The association between AF and all-cause mortality had a significant interaction with the etiology of MR. The presence of AF was associated with 2-year mortality after M-TEER in patients with DMR, independently of clinical and echocardiographic characteristics; however, the association was attenuated in patients with VFMR or AFMR (Central Illustration).
3. Rate of residual MR ≤2+ at 1- and 12-month follow-ups was consistent between patients with and without AF in each MR etiology.
4. Among patients with VFMR, the association between AF and all-cause mortality was modulated by LA volume index.

The development of catheter ablation has markedly changed the management of AF in patients with heart failure. Recent randomized controlled trials demonstrate prognostic benefits of catheter ablation for AF in patients with heart failure.^22^^,^^23^ Based on these results, current guidelines recommend ablation-based rhythm control for AF in patients with heart failure.^24^^,^^25^ Nonetheless, it is important to highlight that these trials did not include patients with severe valvular diseases requiring surgical intervention, including M-TEER. Although AF may adversely affect LA and LV remodeling and negatively affect prognosis, it is uncertain whether AF itself can be still a driver of clinical outcomes in patients with severe MR who have already advanced LA and LV remodeling.

Given the distinct pathophysiological mechanisms, comorbidities, and cardiac function related to MR etiology, it may be necessary to examine the clinical implication of AF separately in each MR etiology. In a study from the Transcatheter Valve Therapy Registry,^12^ AF was associated with a higher risk of mortality or heart failure hospitalization within 1 year in patients with DMR. The prognostic impact of AF in patients with VFMR, however, remains controversial. A meta-regression analysis consisting of 15 observational studies indicated a relationship between AF and mortality in patients with functional MR undergoing M-TEER,^13^ and a subanalysis from the COAPT (Cardiovascular Outcomes Assessment of the MitraClip Percutaneous Therapy for Heart Failure Patients with Functional Mitral Regurgitation) trial showed that AF was associated with a higher risk of mortality or heart failure hospitalization.^10^ On the other hand, recent reports from real-world multicenter registries of VFMR or AFMR patients undergoing M-TEER imply that the relationship between AF and clinical outcomes may be potentially attenuated by underlying clinical characteristics and cardiac function.^26^^,^^27^

The present study is based on the previous studies and further expand them by showing that the association between AF and mortality had a significant interaction with MR etiology. Although durability of MR reduction by M-TEER up to 12 months was comparable between patients with and without AF, the presence of AF was associated with all-cause mortality after M-TEER in patients with DMR even after adjusting for baseline characteristics and procedural results, which was in line with the previous studies.^4^^,^^12^ The increase in all-cause mortality of DMR patients with AF was mainly due to cardiovascular causes. In contrast, the association was attenuated in patients with VFMR and AFMR. Although the presence of AF was linked to a higher incidence of heart failure hospitalization in patients with VFMR, this association did not remain significant after the multivariable adjustment. These results suggest that the impact of AF on clinical outcomes after M-TEER may be modulated by MR etiology.

The heterogeneous prognosis of AF between MR etiologies may reflect differences in underlying cardiac remodeling and function. In patients with DMR, AF may have a more direct adverse hemodynamic impact by eliminating effective atrial contraction, disrupting atrial-ventricular synchrony, and further compromising forward flow. In contrast, patients with VFMR or AFMR typically present with pronounced ventricular remodeling and substantial preexisting LA enlargement, suggesting a more advanced atrial myopathy.^28^ In this setting, where LA functional impairment is already established, the incremental hemodynamic effects of AF might be limited. Consequently, AF may contribute less independently to prognosis in the functional MR etiologies.

Our finding that the association between AF and clinical outcomes after M-TEER was attenuated in patients with VFMR may not be consistent with the previous studies.^10^^,^^13^ One plausible explanation for this discrepancy may be that the earlier studies lacked sufficient adjustment for the clinical and echocardiographic variables, although patients with AF often have advanced cardiac remodeling and multiple comorbidities. In contrast, we conducted the more comprehensive adjustment for these variables, which could allow us to evaluate the prognostic impact of AF itself. This implies that the prognosis of patients with VFMR or AFMR requiring M-TEER may be more likely driven by underlying cardiac remodeling or comorbidities related to, or predisposing to, AF rather than by AF per se.

LA remodeling may be an important determinant of the prognostic impact of AF in patients with VFMR. Our results showed that AF tended to be associated with a higher risk of 2-year mortality in VFMR patients with lower LA volume index, whereas this association was attenuated in those with more advanced LA enlargement. LA enlargement can occur as a consequence of MR or AF and is often accompanied by impaired LA function,^28^ which could worsen the prognosis. In patients who have severe VFMR and already advanced LA remodeling, the incremental effect of AF on the LA remodeling and prognosis may be limited. Compared with the subanalysis from the COAPT trial,^10^ the VFMR patients included in the present study had a larger LA volume index (85 mL/m^2^ vs 48 mL/m^2^). This difference in the degree of LA remodeling may be another possible explanation for the discrepancy in the prognostic impact of AF in patients with VFMR between our study and the previous report. Nonetheless, it should be emphasized that our study population may be more representative of real-world clinical practice. LA volume index may be a useful marker in estimating the prognostic implication of AF in patients with VFMR.

Our findings should be interpreted with caution because the diagnosis of AF was not dependent on ambulatory monitoring or any prespecified protocols and AF burden was not systematically quantified in this multicenter registry. AF burden, as well as the presence of subclinical AF, is increasingly recognized as an important determinant of cardiac remodeling and clinical outcomes.^29^^,^^30^ The results of our sensitivity analyses in which patients were categorized according to subtypes of AF or atrial rhythm on the electrocardiogram were consistent with the main analysis. Nonetheless, the inability to evaluate AF burden or subclinical AF episodes in the present study may potentially obscure the true magnitude of AF-related risk, particularly in patients with VFMR or AFMR. Future studies incorporating continuous or protocolized rhythm monitoring are needed to more clearly delineate the prognostic impact of AF and its burden after M-TEER.

The present work would assist with the management of AF in patients with MR who undergo M-TEER. Given the prognostic impact of AF, continued management of AF would remain important even after M-TEER in patients with DMR. Patients with DMR may need more intensive rhythm surveillance to detect AF. If patients with DMR have AF, closer longitudinal follow-up and stronger consideration of rhythm-control strategies, including catheter ablation, may be warranted. On the other hand, the rhythm control of AF may come “too late” in patients with VFMR or AFMR who require the intervention for MR, because the cardiac remodeling and dysfunction have already advanced. Earlier identification and intervention of AF before the progression of functional MR might be important to optimize long-term outcomes of these patients. Nonetheless, our findings should be interpreted as hypothesis-generating. Prospective studies are needed to examine the clinical benefits of ablation-based rhythm control for AF in patients with MR requiring M-TEER.

### Study limitations

First, this is a post hoc analysis using data from a prospective multicenter registry. Although we conducted a comprehensive adjustment, several unmeasured confounders might affect our results. Second, the data of management of AF, including medication and a prior history of catheter ablation, were lacking in the registry data. Finally, LA functional parameters, such as LA strain or minimum LA volume, were not evaluated in the present study, although we examined the interaction of the association between AF and mortality with LA volume index. LA function may not always correspond to the LA size and could provide further information on the clinical implication of AF.^31^ Future studies are needed to examine the impact of AF on the prognosis after M-TEER according to the LA function.

## Conclusions

AF was frequently present in patients undergoing M-TEER. The association between AF and all-cause mortality had a significant interaction with the etiology of MR. AF was associated with a higher risk of all-cause mortality after M-TEER in patients with DMR, whereas this association was attenuated in patients with VFMR and AFMR. The prognostic effect of AF in patients undergoing M-TEER may be modulated by MR etiology and underlying LA remodeling.

## Funding Support and Author Disclosures

The OCEAN-Mitral (Optimized CathEter vAlvular iNtervention–Mitral) registry is part of the OCEAN-SHD (Optimized CathEter vAlvular iNtervention Structural Heart Disease) registry and is supported by Edwards Lifesciences, Medtronic Japan, Boston Scientific, Abbott Medical Japan, and Daiichi-Sankyo Company. Drs Saji, Kubo, Izumo, Kuwata, Watanabe, and Amaki are clinical proctors of transcatheter edge-to-edge repair for Abbott Medical and have received consultant fees from Abbott Medical. Drs Asami and Kodama are clinical proctors of transcatheter edge-to-edge repair for Abbott Medical and have received speaker fees from Abbott Medical. Dr Yamamoto is a clinical proctor of transcatheter edge-to-edge repair for Abbott Medical and has received lecture fees from Abbott Medical. Dr Yamaguchi is a clinical proctor of transcatheter edge-to-edge repair for Abbott Medical and has received a lecture fee and scholarship donation from Abbott Medical. Dr Ohno is a clinical proctor of transcatheter edge-to-edge repair for Abbott Medical, and has received consultant, advisor, and speaker fees from Abbott Medical. Drs Enta, Shirai, Mizuno, Ueno, Bota, and Hayashida are clinical proctors of transcatheter edge-to-edge repair for Abbott Medical. All other authors have reported that they have no relationships relevant to the contents of this paper to disclose.
