# Supplementary material for: Atrial Fibrillation and Clinical Outcomes Following Mitral Transcatheter Edge-to-Edge Repair According to Mitral Regurgitation Etiology
Source: JACC Asia. 2026 May 6;6(8):1370–81. doi: 10.1016/j.jacasi.2026.02.028 (PMC13458923; doi:10.1016/j.jacasi.2026.02.028)
Supplement: Supplementary file 1 [file mmc1.docx]

**SUPPLEMENTAL MATERIAL**

**Atrial fibrillation and clinical outcomes following mitral transcatheter edge-to-edge repair according to mitral regurgitation etiology**

**Running title: AF and MR etiology in M-TEER**

**Authors:** Tetsu Tanaka, MD^1^; Masaki Izumo, MD^1^; Shingo Kuwata, MD^1^; Taishi Okuno, MD^1^; Yoshihiro J Akashi, MD^1^; Masanori Yamamoto,MD^2,3,4^; Shunsuke Kubo, MD^5^; Mike Saji , MD^6,7^; Yuki Izumi, MD^6^; Yusuke Enta, MD^8^; Shinichi Shirai, MD^9^; Shingo Mizuno, MD^10^; Yusuke Watanabe, MD^11^; Makoto Amaki, MD^12^; Kazuhisa Kodama, MD^13^; Junichi Yamaguchi , MD^14^; Toru Naganuma , MD^15^; Hiroki Bota, MD^16^; Yohei Ohno, MD^17^; Masahiko Asami, MD^18^; Daisuke Hachinohe, MD^19^; Masahiro Yamawaki, MD^20^; Hiroshi Ueno, MD^21^; Kazuki Mizutani, MD^22^; Toshiaki Otsuka, MD^23^; Kentaro Hayashida, MD^24^; on behalf of the OCEAN-Mitral Investigators

**Supplemental Method.** Multiple imputation

**Supplemental Table 1.** Percentage of missing values in the variables included in the multivariable Cox models

**Supplemental Table 2.** Pattern of variables with missing values

**Supplemental Table 3.** Variables related to the probability of missingness

**Supplemental Table 4.** Complete-case analysis of multivariable Cox regression models

**Supplemental Figure 1.** Study flowchart

**Supplemental Figure 2.** Incidence of stroke and transient ischemic attack

**Supplemental Figure 3.** Severity of mitral regurgitation after mitral transcatheter edge-to-edge repair

**Supplemental Figure 4.** Two-year mortality according to subtypes of atrial fibrillation

**Supplemental Figure 5.** Two-year mortality according to atrial rhythm

**Supplement Methods**

*Multiple imputation*

Missing data were assessed for the variables included in the multivariable Cox models, and the pattern of missing data were summarized in ***Supplemental Tables 1 and 2***. Little’s MCAR test indicated that the data were not missing completely at random (p <0.001). Therefore, we performed multiple imputation under the missing at random (MAR) assumption.

To assess the plausibility of the missing at random assumption, we (1) explored associations between the probability of missing data and observed variables and (2) performed complete-case analyses as a sensitivity analysis.

Logistic regression analyses of the probability of missingness were conducted for variables with a missing rate ≥5%, and these analyses showed that the probability of missing data was associated with some observed baseline parameters (***Supplemental Table 3***).

The imputation models included all variables listed in Table 1, as well as the forementioned variables, and outcome variables to preserve associations with the outcomes. Continuous variables were imputed using linear regression models, binary variables using logistic regression models, and ordinal variables using ordered logistic regression models.

We conducted multiple imputation by chained equations (MICE) because it allows flexible modeling of variables with different distributions (continuous and categorical) and is appropriate under the missing-at-random assumption, which was considered reasonable in the present registry-based cohort. Twenty imputed datasets were generated with 10 iterations per imputation. Cox proportional hazards regression analyses were performed separately in each imputed dataset, and parameter estimates were pooled using Rubin’s rules to account for uncertainty due to missing data.

Diagnostics of the imputation procedure included checks for implausible imputed values, comparisons of the distributions of imputed and observed data, and assessment of the stability of effect estimates across imputations. No implausible values were observed, and the distributions of imputed values were comparable to those of the observed data.

**Supplemental Table 1.** Percentage of missing values in the variables included in the multivariable Cox models

| Variables | N=3764 |
| --- | --- |
| Body mass index | 3 (0.08) |
| Clinical frailty scale | 132 (3.5) |
| Prior HF hospitalization | 10 (0.3) |
| eGFR | 22 (0.6) |
| LVEF | 4 (0.1) |
| LVEDVi | 165 (4.4) |
| TAPSE | 710 (18.9) |
| LA volume index | 144 (3.8) |
| SPAP | 584 (15.5) |
| Values are number with %.  Abbreviations: HF, heart failure; eGFR, estimated glomerular filtration rate; LVEF, left-ventricular ejection fraction; LVEDVi, left ventricular end-diastolic volume index; TAPSE, tricuspid annular plane systolic excursion; LA, left atrium; SPAP, systolic pulmonary artery pressure. | |

**Supplemental Table 2.** Pattern of variables with missing values

| Variables with missing values | N=3764 |
| --- | --- |
| None | 2372 (63.0) |
| TAPSE | 442 (11.7) |
| SPAP | 414 (11.0) |
| TAPSE+SPAP | 102 (2.7) |
| Clinical frailty scale | 87 (2.3) |
| TAPSE+LA volume index | 61 (1.6) |
| LVEDVi | 46 (1.2) |
| TAPSE+LVEDVi | 35 (0.9) |
| LA volume index | 30 (0.8) |
| Clinical frailty scale + LVEDVi | 27 (0.7) |
| Values are number with %.  Abbreviations: LVEDVi, left ventricular end-diastolic volume index; TAPSE, tricuspid annular plane systolic excursion; LA, left atrium; SPAP, systolic pulmonary artery pressure. | |

**Supplemental Table 3.** Variables related to the probability of missingness

| Parameters with a missing rate ≥5% | Variables | β coefficients | 95%CI | p-value |
| --- | --- | --- | --- | --- |
| TAPSE |  |  |  |  |
|  | Clinical frailty scale | 0.22 | 0.006–0.45 | 0.044 |
|  | Atrial fibrillation | 0.28 | 0.02–0.54 | 0.037 |
|  | Prior stroke | 0.31 | 0.01–0.62 | 0.042 |
|  | LVEDVi | -0.41 | -0.67 to -0.15 | 0.002 |
|  | Use of anticoagulants | -0.95 | -1.21 to -0.69 | 0.001 |
| SPAP |  |  |  |  |
|  | Hypertension | 0.33 | 0.09–0.57 | 0.008 |
|  | LVEF | 0.42 | 0.15–0.69 | 0.002 |
|  | Use of anticoagulants | -0.42 | -0.68 to -0.15 | 0.002 |
|  | COPD | -0.44 | -0.88 to -0.01 | 0.047 |
|  | HF hospitalization | 0.59 | 0.31–0.87 | 0.001 |
|  | Use of loop diuretics | -0.41 | -0.67 to -0.15 | 0.002 |
|  | TAPSE | -0.28 | -0.51 to -0.06 | 0.014 |
| Abbreviations: LVEDVi, left ventricular end-diastolic volume index; TAPSE, tricuspid annular plane systolic excursion; HF, heart failure; SPAP, systolic pulmonary artery pressure; COPD, chronic obstructive pulmonary disease. | | | | |

**Supplemental Table 4.** Complete-case analysis of multivariable Cox regression models

|  | Adjusted HR | 95%CI | p value |
| --- | --- | --- | --- |
| DMR |  |  |  |
| All-cause mortality | 1.62 | 0.76–3.44 | 0.21 |
| CV mortality | 1.52 | 0.57–4.05 | 0.39 |
| HF hospitalization | 1.02 | 0.54–1.91 | 0.95 |
| VFMR |  |  |  |
| All-cause mortality | 0.95 | 0.67–1.35 | 0.78 |
| CV mortality | 0.89 | 0.61–1.29 | 0.54 |
| HF hospitalization | 0.93 | 0.70–1.25 | 0.64 |
| AFMR |  |  |  |
| All-cause mortality | 0.95 | 0.70–1.29 | 0.76 |
| CV mortality | 0.75 | 0.16–3.64 | 0.72 |
| HF hospitalization | 0.42 | 0.17–1.00 | 0.05 |
| Abbreviations: DMR, degenerative mitral regurgitation; VFMR, ventricular functional mitral regurgitation; AFMR, atrial functional mitral regurgitation; CV, cardiovascular; HF, heart failure. | | | |

**Supplemental Figure 1.** Study flowchart

Legends: M-TEER, mitral transcatheter edge-to-edge repair; DMR, degenerative mitral regurgitation; VFMR, ventricular functional mitral regurgitation; AFMR, atrial functional mitral regurgitation; AF, atrial fibrillation.

**Supplemental Figure 2.** Incidence of stroke and transient ischemic attack

Incidence of stroke and transient ischemic attack according to atrial fibrillation (AF) in patients with degenerative mitral regurgitation (A), ventricular functional mitral regurgitation (B), and atrial functional mitral regurgitation (C).

**Supplemental Figure 3.** Severity of mitral regurgitation after mitral transcatheter edge-to-edge repair

Legends: Rate of moderate or less mitral regurgitation (MR) at baseline, discharge, and one- and twelve-month follow-ups in patients with degenerative MR (A), ventricular functional MR (B), and atrial functional MR (C).

AF = atrial fibrillation.

**Supplemental Figure 4.** Two-year mortality according to subtypes of atrial fibrillation

Incidence of two-year mortality according to subtypes of atrial fibrillation (AF) in patients with degenerative mitral regurgitation (A), ventricular functional mitral regurgitation (B), and atrial functional mitral regurgitation (C).

**Supplemental Figure 5.** Two-year mortality according to atrial rhythm

Incidence of two-year mortality according to atrial rhythms, such as sinus rhythm, atrial fibrillation (AF), or atrial pacing, in patients with degenerative mitral regurgitation (A), ventricular functional mitral regurgitation (B), and atrial functional mitral regurgitation (C).
